# A 64 × 64 GaN Micro LED Monolithic Display Array: Fabrication and Light Crosstalk Analysis

**DOI:** 10.3390/mi16020207

**Published:** 2025-02-11

**Authors:** Yang Xiao, Yuan Meng, Xiaoyu Feng, Longzhen He, Philip Shields, Sean Lee, Yanqin Wang, Zhifang Wang, Pingfan Ning, Hongwei Liu

**Affiliations:** 1 Tianjin Key Laboratory of Optoelectronic Detection Technology and System, School of Electronics and Information Engineering, Tiangong University, Tianjin 300387, China; 2 Department of Electronic and Electrical Engineering, University of Bath, Bath BA2 7AY, UK; 3 Chip R&D Department, Sanan Optoelectronics Co., Ltd., Tianjin 300392, China; 4 Analytical & Testing Center, Tiangong University, Tianjin 300387, China

**Keywords:** micro light-emitting diode (LED), monolithic display array, photonic crystal, focused ion beam (FIB), optical crosstalk

## Abstract

Monolithic micro LED display arrays show potential for application in small-area display modules, such as augmented reality (AR) displays. Due to the short distance between micro LEDs and the monolithic transparent substrate, a light crosstalk phenomenon exists between adjacent micro LED pixels, decreasing the array’s display definition. In this paper, a 64 × 64 GaN micro LED monolithic display array was fabricated on a silicon-based drive circuit. The micro LED size was 20 μm × 20 μm, and the pitch between micro LEDs was 28 μm. To suppress the optical crosstalk between adjacent micro LEDs in the array, we etched a photonic crystal structure using a focused ion beam (FIB) on the micro LED sapphire substrate. Measurements of the micro LED nearfield electroluminescence (EL) and finite element method (FEM) calculations demonstrated that the light expansion was confined in the photonic crystal micro LED with a thinner substrate. The presented work provides references regarding the fabrication of monolithic micro LED arrays and the control of crosstalk in displays.

## 1. Introduction

Micro LEDs are a promising candidate for next-generation display technology, offering better contrast and saturation than a liquid crystal display panel [1]. Compared to organic light-emitting diodes (OLEDs), III-nitride-based micro LEDs exhibit higher resolution and reliability [2,3,4].

However, there are also some challenges for the micro LED display application, such as its high wafer cost. Therefore, a micro LED panel in traditional large-area TV and monitor displays is limited by the high cost of its fabrication. Consequently, large-area display panels are mainly limited to commercial display scenarios [5,6,7]. For small-area display applications, such as augmented reality (AR) and wearable display devices, micro LEDs are currently the most promising solution [8,9,10,11]. In these small-area display devices, the micro LED pixels are mainly used as discrete devices or monolithic arrays. Another challenge is low mass transfer efficiency. The discrete micro LEDs need to be transferred from the wafer substrate and relocated to the drive circuits [12,13,14,15,16,17]. This mass transfer process may cause a transfer loss in the micro LED display panel. For a monolithic micro LED display array [18], micro LEDs can be transferred with their original monolithic substrate, which has a much higher yield than the discrete micro LED mass transfer method. Importantly, the monolithic micro LED arrays are fabricated on the drive circuits using a flip-chip process so that there are no LED electrodes blocking the light extraction [19,20,21]. This approach can also provide a high-thermal conductivity path for LED heat dissipation [22]. The monolithic micro LED array can be used for a monochromatic display or combined with a color-mixing prism to project an RGB display.

In this work, we fabricated a GaN 64 × 64 micro LED monolithic display array on a passive drive circuit with a flip-chip aligner. The sapphire substrate of the GaN micro LED array remained as a support frame and as display windows. The size of a single micro LED pixel within the monolithic array was 20 μm × 20 μm, and its electro-optic characteristics were measured.

We found that the micro LED light power distribution covered the adjacent pixel positions and was caused by the micro size of the LEDs and the waveguiding of the sapphire transparent substrate, leading to optical crosstalk between the adjacent pixels. The smaller the size and separation of the micro LEDs, the more serious this problem became, leading to a decrease in the display resolution.

There are several methods that can be used to weaken the crosstalk between the display pixels, such as substrate removal and wafer bonding [23,24]. These methods require micro LED lift-off from the transparent substrate, which changes the monolithic array to discrete micro LEDs and may cause GaN LED damage. Reducing the sapphire substrate thickness and implementing a light extraction structure [25,26,27,28,29] could be an alternative route by which to reduce micro LED light crosstalk.

In this paper, a sapphire substrate photonic crystal structure was employed to verify the light crosstalk effect. After monolithic micro LED array chip fabrication and wafer dicing, it is difficult to apply photolithography masks and dry etching techniques to produce micro/nano patterns on the surface of the sapphire substrate. Therefore, we used FIB technology to process the photonic crystal structure in the substrate area. Through the graphic, coordinates are established by the FIB system, and the relative position of the photonic crystal area and the micro LED pixel area can be accurately controlled.

## 2. Experiments

**Figure 1:**
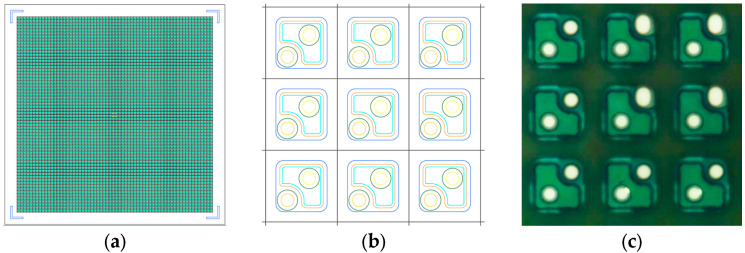
(**a**) Schematic of 64 × 64 monolithic display array; (**b**) internal view. (**c**) A local 500× microscope photo of the display array.

In this work, a GaN 64 × 64 micro LED monolithic display array was bonded onto a passive drive circuit using a flip-chip aligner. The display chip size of the 64 × 64 micro LED array, as shown in Figure 1, was 2016 μm × 2016 μm. The size of the micro LEDs in the display array was 20 μm × 20 μm, and the pitch of the micro LEDs was 28 μm.

The micro LED wafer layers from top to bottom consisted of 400 nm P-GaN, 180 nm 10-period GaN/InGaN multi-quantum wells, 1.5 μm N-GaN, a 3 μm undoped GaN buffer layer, and 150 μm of sapphire substrate. The array quantum well emission peak wavelength was 460 nm. The micro LED P, N electrode Au pads were situated on the top of the device. The N pad (thickness = 0.5 μm) was fabricated on the N-GaN following an ICP (inductive coupled plasma) etch through the P-layer, and the P electrode pad (thickness = 0.5 μm) was integrated into the 1 μm ITO transparent current spreading layer of the P-GaN. The P, N metal layer underwent rapid thermal annealing (@ 450 °C, 10 min). Then, the SiNx layer isolated and protected the P and N zone mesa. The micro LED P, N pad diameters were 5 μm.

**Figure 2:**
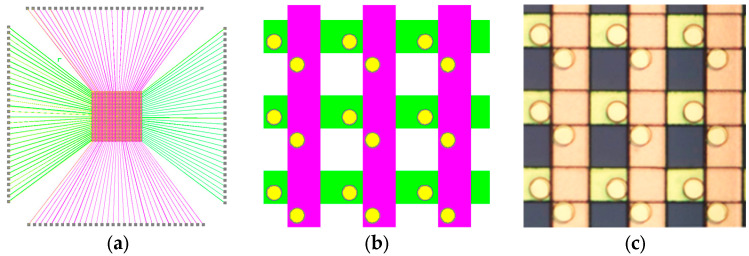
Schematic of the 64 × 64 micro LED display array drive circuit on Si substrate. (**a**) Overall diagram of the drive circuit, (**b**) the internal P, N electrode for micro LED metal contact, and (**c**) a local 500× microscope photo of the display array drive circuit.

The drive circuit structure is shown in Figure 2, including the high-resistivity Si substrate, the Ti/Au row line for the LED anode, the Ti/Au column line for the LED cathode, and Au pads for the LED P, N electrode connection. As shown in Figure 2a, the P, N electrode lines are extruded to the 100 μm × 100 μm connection pad at the edge of the silicon substrate. In order to save substrate area space and facilitate the gold wire bonding, P-type odd-numbered electrode pads are arranged on the upper boundary of the substrate, and even-numbered P electrode pads are arranged on the lower boundary. Similarly, N-type odd electrode pads are arranged on the left side of the chip, while even N electrodes pads are on the right.

The 64 × 64 P, N Ti/Au electrode line thickness is 0.1/0.3 μm, which are isolated by a 0.4 μm thickness SiNx layer. The heights of the P, N Au rods for the electrode connection are 1 μm and 2.6 μm, respectively, and their height difference is used to compensate for the thickness difference caused by PN wafer etching on the micro LED chips. Every electrode line width is 12 μm, and the diameter of the P, N rods is 5 μm.

**Figure 3:**
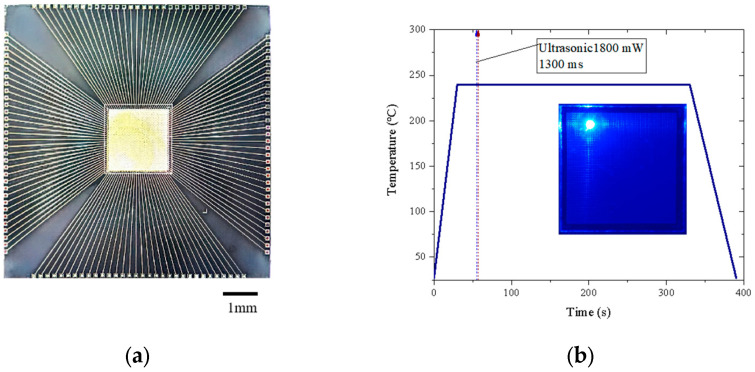
(**a**) The monolithic micro LED array; (**b**) its flip-chip bonding temperature and ultrasonic welding power; the inset photo shows a micro LED pixel lighting at a 12.5 A/cm^2^ forward bias current.

The micro LED display array was fabricated on the Si substrate with integrated drive circuits using a flip-chip bonding method. The fabricated micro LED display array is shown in Figure 3a. This process was completed using a Fineplacer 2000 (Finetech, Berlin, Germany), and the bonding temperature curve is illustrated in Figure 3b. The flip-chip bonding pressure was 15 N, and an 1800 mW ultrasonic power was applied to the bonding chips at 25 s and maintained for 1300 ms.

After flip chip bonding, the micro LEDs in the display array can be driven by the bias voltage through the Si drive circuits row and column pads. The micro LED pixel working microscope photo (at a 12.5 A/cm^2^ forward bias current) is shown in the inset of Figure 3b. It is observed that when a single pixel works, the adjacent pixels and areas also become brighter because of the effect of optical crosstalk, which will blur the array display image and reduce resolution. The smaller the chip size, the lower the display resolution, as a result of crosstalk.

**Figure 4:**
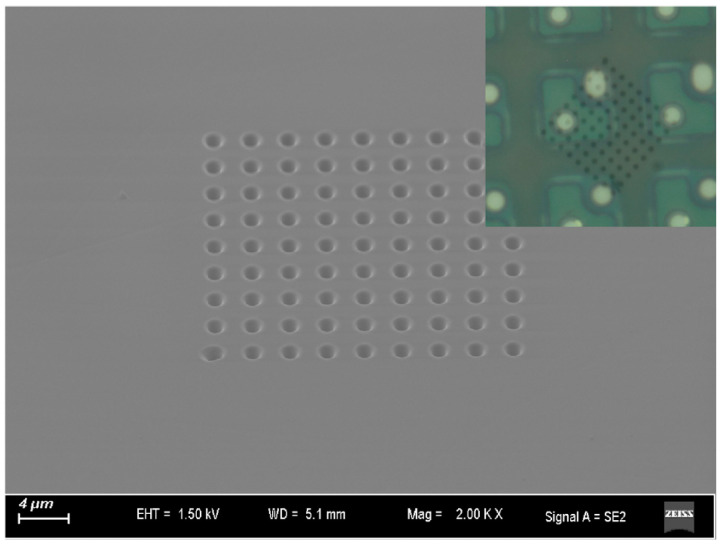
An SEM image of the photonic crystal structure fabricated on a micro LED on the sapphire substrate. The inset microscope photo shows the relative position of the photonic crystal and the micro LED pixel.

In this work, we half-covered a micro LED pixel with a photonic crystal to analyze its influence on the micro LED light output surface light transmission. The photonic crystal was etched into the sapphire substrate surface so that there was no danger of the process damaging the micro LED. To avoid the differences in micro LEDs optoelectronic characteristics, we chose a single micro LED pixel in the display array as the processing and analyzing device. The photonic crystal area referred to the micro LED P, N electrodes diagonal direction, which ensured that half of the micro LED light output surface was covered by photonic crystal air holes and the other half with the original sapphire substrate (as shown in the inset microscope image of Figure 4). The photonic crystal fabrication processing was as follows.

After the micro LED array flip chip bonding, we fabricated a photonic crystal air hole array on the rear side of the micro LED sapphire substrate with a Focused Ion Beam (FIB) and Electron Beam Microscope system (Zeiss, Baden-Württemberg, Germany). Before device fabrication, we employed the Rigorous Coupled-Wave Analysis (RCWA) algorithm to calculate the parameters of the sapphire photonic crystal structure. The parameters (square lattice constant = 3 μm, duty cycle = 50%) were chosen with an optimal band structure and high transmission for the micro LED central wavelength of 460 nm.

Due to the photonic crystal being etched into the sapphire substrate, the SEM system of the Cross Beam 550 could not simultaneously observe and locate the micro LED pixel on the reverse side. Consequently, it was necessary to align the sapphire substrate rear side photonic crystal area and the front side of the micro LED pixel area before the fabrication of the photonic crystal air holes.

Therefore, we first located the micro LED pixel on the rear side of the sapphire substrate with an optical microscope and made pixel diagonal position marks on the rear side of the sapphire substrate corresponding to the pixel area of the micro LED. With the substrate marks, we set up the FIB coordinate system to locate the photonic crystal area before starting the fabrication. During the creation of the photonic crystal air holes, the FIB Gallium^+^ ion beam energy was 30 keV and the beam current density was 700 pA.

The final 9 × 9 photonic crystal air holes on the sapphire substrate SEM image are shown in Figure 4. The lattice constant of the photonic crystal is 3 μm, the radius of the air holes is 0.75 μm, and the depth of the air holes is 2 μm. A microscope image of the micro LED pixel through the photonic crystal sapphire substrate is shown in the inset of Figure 4. Only half of the central micro LED substrate was covered by the photonic crystal structure. In this work, the FIB technology was used for the convenience of processing photonic crystal air holes on an approximately 2 mm × 2 mm micro LED display array. Furthermore, the advantage of FIB processing was that the processing area and processing design were flexible and varied, but the fabrication time was about 36 min, and the fabrication efficiency was low.

## 3. Results and Discussion

**Figure 5:**
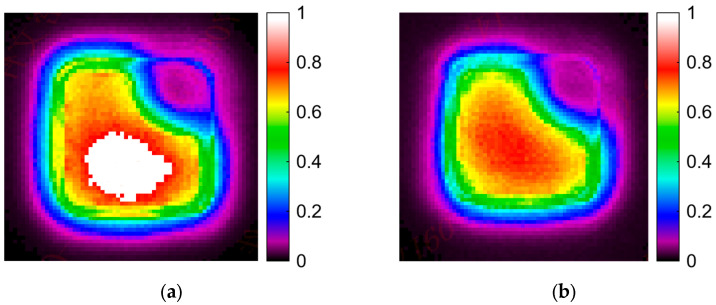
The light emission profile of micro LED display array pixels measured using a laser beam profiler (at a 12.5 A/cm^2^ current density). (**a**) Micro LED with diagonal covered by a photonic crystal structure. (**b**) Micro LED without a photonic crystal structure.

The light profile of the micro LED pixels from the sapphire substrate side was observed using a laser beam profiler (Zolix, Beijing, China). The optical power distribution of the micro LEDs shows that the light intensity increases in the photonic crystal-covered area (as shown in Figure 5a). This means that more light was extracted from the sapphire substrate, and the light wave guide modes of the substrate were destroyed, which suppresses the optical crosstalk between the pixels of the micro LED display array.

**Figure 6:**
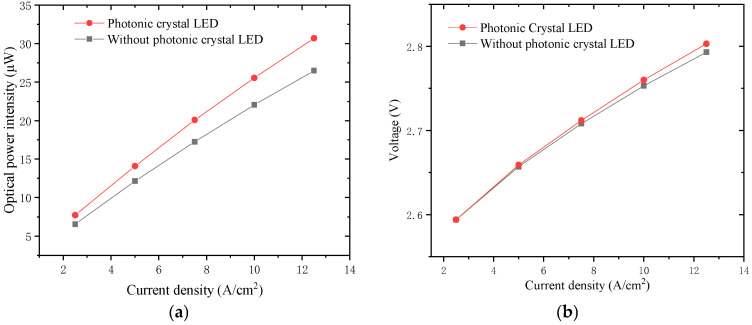
The light output power (**a**) and I-V curve (**b**) of micro LED display array pixels tested by electroluminescence measurement.

Furthermore, the electroluminescence (EL) characteristics of the micro LED pixels were tested with a Laser Beam Profiler (Zolix, Beijing, China) and a Keithley^TM^ 4200A-SCS Parameter Analyzer (Tektronix, Beaverton, OR, USA). We compared the half-photonic-crystal-covered micro LED pixel (photonic crystal LED) and the pixel without photonic crystal (without photonic crystal LED). The light output from the photonic crystal LED, under a current bias ranging from 2.5 A/cm^2^ to 12.5 A/cm^2^, demonstrated a nearly 15% increase in average output power, as shown in Figure 6. The EL test results prove that the photonic crystal structure can improve the micro LED light extraction efficiency. If photonic, crystal, full-covered micro LED samples were used in EL measurements, the light output power would be further enhanced.

**Figure 7:**
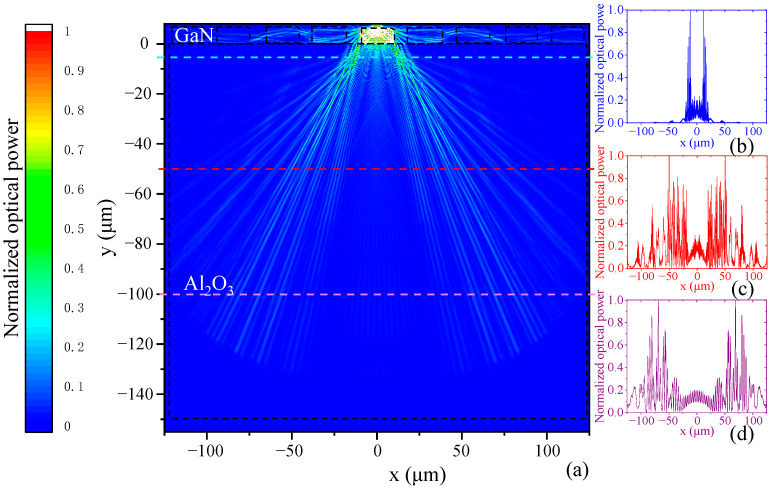
(**a**) The calculated light power distribution in the GaN micro LED array and sapphire substrate. The right insets show the normalized light power distribution in the sapphire substrate at distances of (**b**) 5 μm, (**c**) 50 μm, and (**d**) 150 μm from the micro LED.

In order to analyze the optical crosstalk effect in the micro LED array, we set up a light transmission model using Lumerical^TM^ FDTD Solutions software (ANSYS 2024 R2, Ansys, Canonsburg, PA, USA). In our model, GaN LEDs with a length of 20 μm and a height 6 μm thick were located on a 150 μm thick sapphire substrate. The period of micro LEDs was 28 μm, and a 460 nm wavelength dipole light source was set in the middle of the GaN LED to simulate the light emission from the micro LED pixels. Figure 7 shows the calculated optical power distribution of one micro LED pixel working in the monolithic display array.

From the calculated result (Figure 7), we can see that the micro LED light emission expands at the boundary of GaN and sapphire, and the crosstalk is mainly caused by the sapphire substrate, which creates a light wave guide between the micro LED pixels. To suppress the micro LED light expansion in the sapphire substrate, we could reduce the thickness of the substrate and enhance the light transmission of the substrate light output surface.

From the above measurements and analysis, it can be determined that the light crosstalk effect of the monolithic micro LED array is affected by the sapphire substrate light transmission and thickness (Figure 4, Figure 5, Figure 6 and Figure 7). We built a 3 × 3 micro LED array model to analyze the light crosstalk in the monolithic micro LED array with FDTD Solutions software, recording the light power distribution of the micro LED array light output surface.

To facilitate the statistical evaluation of the impact of micro LED array structures on optical crosstalk characteristics and to ensure the symmetry of the light distribution in a 3 × 3 symmetric structure model, we positioned a dipole source at the center of the micro LED array model. The dipole source radiated in the positive *z*-axis direction. The boundary conditions of the array were set to the PML (Perfectly Matched Layer) to minimize reflections and ensure accurate results.

**Figure 8:**
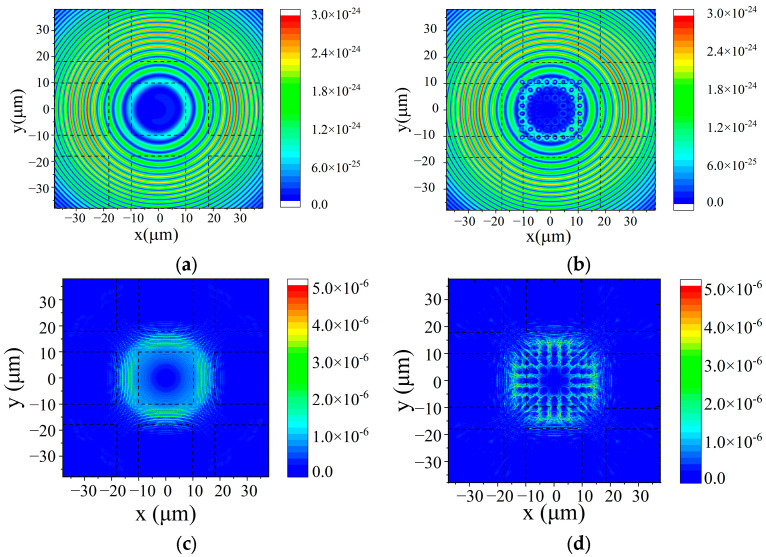
The light power simulation at the 3 × 3 micro LED array sapphire output surface. (Note: Dashed lines are micro LED positions). (**a**) 150 μm sapphire substrate micro LEDs without a photonic crystal structure. (**b**) 150 μm sapphire substrate micro LEDs with a photonic crystal structure. (**c**) 5 μm sapphire substrate micro LEDs without a photonic crystal structure. (**d**) 5 μm sapphire substrate micro LEDs with a photonic crystal structure.

In this model, 20 μm × 20 μm × 6 μm 3 × 3 GaN arrays were placed on the sapphire substrate. A 460 nm wavelength dipole light source was placed at the center of the 3 × 3 GaN micro LED array to simulate light emission from the LED quantum well. And a photonic crystal array was added on the substrate of the central micro LED. The square photonic crystal period was 3 μm, the duty ratio was 0.5, and the depth of the air hole was 2 μm; the same as the photonic crystal fabricated by the FIB in this work. The difference was that the simulated photonic crystal area covers the full dimensions of the central micro LED rather than just half. The thickness of the micro LED sapphire substrate was reduced to 5 μm. For comparison, the light power distribution of the micro LED array at the sapphire output surface with an original 150 μm thick substrate was also calculated (as shown in Figure 8).

The light power distribution from the sapphire substrate showed that the original 150 μm substrate expanded more light from the central LED to other pixels, whereas the light distribution from the thinner substrate was concentrated in the central micro LED area. In addition, the photonic crystal structure significantly increases the maximum value of the light power.

We defined the light expansion factor $F_{Expansion}$ to evaluate the expansion characteristics of micro LED pixel light emission at the sapphire output surface.

(1) $$F_{Expansion}=\frac{E_{Surrounding}}{E_{Central}}$$

**Figure 9:**
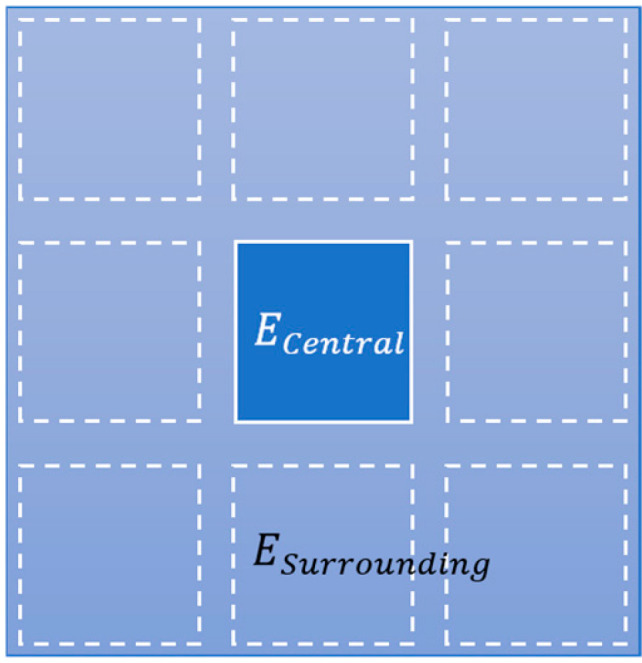
The calculated light expansion factor based on the light power distribution. (Note: Solid line and dashed lines are central and surrounding micro LED positions).

In Equation (1), $E_{Central}$ represents the mean value of the central micro LED’s light power and $E_{Surrounding}$ denotes the power outside of the central micro LED (as shown in Figure 9). The values of the central power, surrounding power, and expansion factor determined from simulations of the micro LED array light power distribution (Figure 8) are shown in Table 1.

For the micro LED with a substrate thickness of 150 μm (Figure 8a), the light expansion is obvious and $F_{Expansion}$ is 3.9609. As for the micro LEDs with photonic crystal structures (Figure 8b), the central light power is enhanced by the photonic crystal and $F_{Expansion}$ is 3.7189.

The calculated photonic crystal central light power ($E_{Central}$) of the 150 μm substrate (Table 1 (a,b)) shows a 2.8% enhancement, which is lower than the Figure 6 experiment measurements. The primary cause of this issue is the use of a single central dipole source in our model to calculate the optical crosstalk of the device. The strong confinement effect of GaN and sapphire materials on the central dipole further aggravates the problem. In contrast, the photonic crystal structure in the actual tested micro LED is fabricated by focused ion beam (FIB) etching, resulting in rough air holes. The rough air hole’s strong light scattering disrupts the light reflection and improves the light extraction efficiency.

For the micro LEDs with a substrate thickness of 5 μm (Figure 8c,d), the light expansion parameters are decreased to around 0.3. The thinner substrate significantly limits light power expansion. Comparing Figure 8c,d, the photonic crystal structure in Figure 8d increases the uniformity of the light power in the central micro LED surface and compresses the surrounding light power. The average intensity of the central light power with a photonic crystal is reduced from that shown in Figure 8c, but the light expansion parameter of Figure 8d is the lowest ($F_{Expansion}$ = 0.3051).

Through the above analysis, we can conclude that the $F_{Expansion}$ parameter can effectively measure the single micro LED pixel light expansion. However, for a multi-pixel display array, the crosstalk between pixels is also influenced by the pixel size and pitch. Therefore, we used convolutional evaluation methods [30,31] to analyze the light distribution between multiple pixels.

We define the micro LED array’s light power distribution matrix as $Image\left[x,y\right]$, and the convolution kernel function is represented by $Filter\left[u,v\right]$, which is an all-1 matrix with a size of the light distribution data from a single micro LED. The convolution step size is set as the micro LED data period. After the convolutional calculation, the $Image\left[x,y\right]$ can be simplified as a matrix $Conv\left[i,j\right]$ in Equation (2).

(2) $$Conv\left[i,j\right]=\overset{i,j=0,1,2}{\underset{u,v}{\sum }}Image\left[i\cdot period+u,j\cdot period+v\right]\cdot Filter\left[u,v\right]$$

The elements of $Conv\left[i,j\right]$ are the sum of light power within the range of micro LED pixels, and the step of the convolution matrix operations is the period of the micro LED array. This operation covers three parameters: micro LED light power distribution, pixel size, and pixel interval, which can be used to measure the cross-correlation between different micro LED pixels.

We define the light crosstalk factor ($F_{C\_T}$) of Equation (3) to describe the light correlations between the different micro LED pixels.

(3) $$F_{C\_T}\frac{\left[k,l\right]}{\left[m,n\right]}=\frac{Conv\left[k,l\right]}{Conv\left[m,n\right]}\left(k,l,m,n\in i,j\right)$$

**Figure 10:**
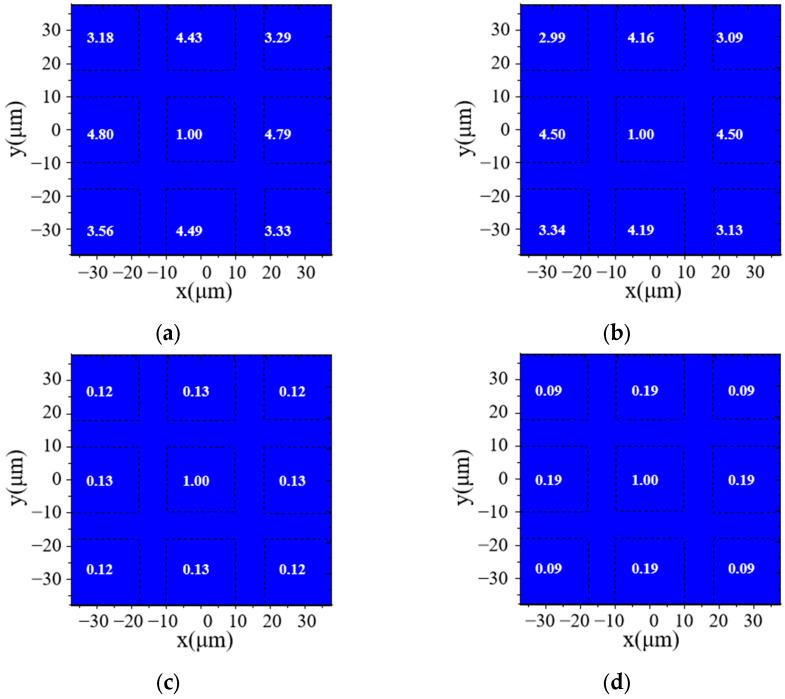
The calculated light crosstalk factor of the central micro LED to the surrounding micro LEDs. (**a**) 150 μm sapphire substrate micro LEDs without a photonic crystal structure. (**b**) 150 μm sapphire substrate micro LEDs with a photonic crystal structure. (**c**) 5 μm sapphire substrate micro LEDs without a photonic crystal structure. (**d**) 5 μm sapphire substrate micro LEDs with a photonic crystal structure.

The calculated light power distribution data from a 3 × 3 micro LED array (Figure 8) are processed using Equation (2), and the $Conv\left[i,j\right]$ is derived. Then, the $F_{C\_T}$ are used to find the cross-correlation between the central LED [1,1] and the other eight surrounding LEDs. The light crosstalk parameter $F_{C\_T}=\frac{\left[i,j\right]}{\left[1,1\right]}$ for each LED is shown in Figure 10.

From Figure 10, the light crosstalk parameters $F_{C\_T}=\frac{\left[i,j\right]}{\left[1,1\right]}$ show the central micro LED light emission crosstalk to other pixels. The light crosstalk parameters of the 150 μm substrate micro LEDs (Figure 10a,b) are higher than 2, and the distribution of light intensity in the surrounding LED positions causes interference in identifying individual pixels. For a 5 μm substrate micro LED array (Figure 10c,d), the center micro LED light distribution has a small impact on the surrounding micro LEDs, and the light crosstalk parameters are around 0.1~0.2.

Comparing Figure 10c,d, the lattice direction of photonic crystals has an impact on the light distribution of the micro LED device. In the horizontal and vertical directions of the micro LED arrays, the light distribution leads to a light crosstalk coefficient of 0.19, while, in the diagonal direction, the light crosstalk coefficient decreases to 0.09.

The $F_{C\_T}$ convolution matrix divides the micro LED display light power distribution map with the pixels’ period, pitch, and size parameters. Therefore, the $F_{C\_T}$ matrix not only contains the pixels’ light power information but also spatial information, which is essential for the pixels’ definition and analyzing inter-pixel optical crosstalk.

Adding 9 × 9 air holes to the photonic crystal had a limited effect compared with the large-scale thinning of the substrate. However, the photonic crystal structure could enhance the maximum light power at a certain wavelength, and in a certain area of the micro LED pixel, the light output power without a photonic crystal area was compressed, which was beneficial to the high definition of the micro LED array, following the theory of our crosstalk parameter $F_{C\_T}$. Therefore, we recommend processing photonic crystal structures corresponding to pixel regions on sapphire substrates while keeping non-pixel regions blank to improve the resolution of the micro LED display array.

## 4. Conclusions

In this work, we designed a monolithic micro LED display array. Our research shows that, as the pixel size and spacing of the micro LEDs decrease, it becomes more difficult to control the pixels’ light output expansion and improve display resolution. However, the micro LED light control is particularly important for monolithic display arrays when they excite RGB phosphors to achieve full-color output pixels.

From the analysis results, both the substrate thinning and photonic crystal structure can suppress optical crosstalk. Thinning the substrate thickness on a large scale (from 150 μm to 5 μm) has a more significant effect on suppressing light crosstalk. However, a thin substrate will reduce the mechanical strength of the micro LED display array, which may lead to array breakage during the flip-chip bonding process. Therefore, the thickness of the sapphire substrate needs a balance between the optical and mechanical characteristics of the display array. In micro LED display applications, these two technologies can be combined to limit the optical crosstalk between the micro LED pixels.

Based on the device structure presented in this article, we propose an evaluation method for LED display array crosstalk. The light expansion factor ($F_{Expansion}$) and light crosstalk factor ($F_{C\_T}$) can be used to judge the correlations of pixel light emission to the surrounding dielectric material and other pixels.

For the flip-chip monolithic micro LED array in this paper, the light output window is the sapphire substrate, and reducing the thickness of the substrate can better limit the pixels’ light crosstalk than a photonic crystal structure. The $F_{Expansion}$ of the micro LED with a 5 μm thick substrate decreases to around 0.3.

Although the electroluminescence (EL) light distribution and finite element method (FEM) calculation prove that a photonic crystal etched into a thick sapphire substrate (150 μm) can enhance the micro LED device light emission, crosstalk, evaluated by $F_{Expansion}$, is not improved. This is because both the micro LED $E_{Central}$ and $E_{Surrounding}$ are increased simultaneously by the scattering of the photonic crystal structure. And the surrounding light emission at the pixel pitch joins the crosstalk effect calculation, which causes an inaccurate cross-light judgement.

Therefore, the crosstalk effect must consider the pixel light emission intensity and distribution and the pixel size, period, and duty cycle. $F_{Expansion}$ is helpful for the analysis of the light expansion characteristics of a single pixel. If we examine the relationship between different pixels or assess the impact of light expansion from a single pixel on the surrounding or interval pixels, the light crosstalk factor, denoted as $F_{C\_T}$, is a more suitable parameter.

## Figures and Tables

**Table 1:** The calculated light expansion factor of the central micro LED of four samples in Figure 8.

Figure 8	Sapphire Substrate	Photonic Crystal Structure	$E_{Surrounding}$	$E_{Central}$	$F_{Expansion}$
a	150 μm	No	1.11319 × 10^−24^	2.81 × 10^−25^	3.9609
b	150 μm	Yes	1.11349 × 10^−24^	2.99 × 10^−25^	3.7189
c	5 μm	No	1.52 × 10^−7^	4.90 × 10^−7^	0.3102
d	5 μm	Yes	1.37 × 10^−7^	4.49 × 10^−7^	0.3051

## Data Availability

The original contributions presented in this study are included in the article. Further inquiries can be directed to the corresponding author(s).
